# The First Medical Peer

**Published:** 1897-01-09

**Authors:** 


### The Hospital

A Journal of

The Medical Sciences and Hospital Administration,

Vol. XXI.—No. 537.] [Jan. 9, 1897.

### THE FIRST MEDICAL PEER.

The announcement in the new year's list of honours of the elevation of Sir Joseph Lister to the peerage was received by the medical profession, as the *Times* said, "with enthusiasm," and by the British nation and the scientific world generally with a feeling of satisfaction hardly less deep and intense. Sir Joseph is the first medical man in Great Britain who has received this distinguishing honour from the hand of our venerated and discriminating sovereign. It is probable that no previous monarch ever thought of raising a physician or surgeon to the peerage. The circumstance marks a distinct change in public feeling, and puts the seal of practically universal approval on the achievements and aspirations of the medical science of the latter half of the nineteenth century.

The personality and work of the new peer are deeply graved upon the mind and feeling of the medical profession. Forty years ago, or longer, the work was begun which has now been crowned with the highest honours an ancient State can bestow. That work has come at length to a completeness, ideal, almost unique, and of a practical importance to the whole world of men and living animals which can never be over-estimated. It has not been given to any scientific worker in any generation of men to achieve a work of more universal and enduring value to all mankind than that achieved by Sir Joseph Lister in his discovery, exposition, demonstration, and successful propagation of the principles of antiseptic medicine and surgery.

Of the new peer, as distinguished from his work, it is impossible for anyone who knows him well to speak without a respect almost amounting to reverence. Twenty-five years ago, in the infancy of antiseptic medicine, the University of Edinburgh and the "Old Infirmary" of that ancient metropolis claimed Joseph Lister as a teacher. His lecture-room was always full; his clinic was always crowded. At the bedside of his hospital patients might have been seen not only all that was accomplished and enthusiastic in the student element, but young graduates in medicine and surgery speaking almost every civilised language under the sun. Frenchmen, Germans, Portuguese, Russians, Americans—from the north and from the south—Hindoos, Parsees; men of all countries and all climes, attracted by his fame, came eagerly to sit at Lister's feet. It is not too much to say that every man among them of competent intelligence, however sceptical on entrance he might be as to antiseptic principles—and sceptiscism was almost universal in those days—went away, after a few weeks or months of clinical work with Lister, as convinced and enthusiastic a Listerian as the great professor himself. If ever a man worked honestly, that man was Joseph Lister; if ever a man worked intelligently and perseveringly, Lister was that man; and if ever a man deserved the honours which his Sovereign has now bestowed, and the acclamations of all cultured and genuinely scientific men, that man is our medical confrère who has just been raised to the peerage.

Our rulers have done wisely in giving this recognition to medical science. The word "progress," so unreal, so false in the mouths of many who, for themselves, so profitably conjure with it, has its true meaning and significance in the higher walks of medicine. It does not mean a mere improvement in the principles and details of the art of healing. It means a practical victory and conquest over nature by man; that and nothing else. Now the greatest of all the aims of civilisation is the acquisition of natural knowledge, the conquest and subdual of nature to the service and happiness of man. The State, therefore, has done well; well for a "progress" which is really worthy of the name, in placing the laurel on Sir Joseph Lister's brow. It is for medicine to recognise this; and to recognise that it is the most illustrious of all British Sovereigns, not excepting great Elizabeth herself, who has done us this act of what we have been accustomed to think tardy and long-delayed justice. But justice has at length been done; and our loyal recognition will make us forget the tedious delay.

A word more may be said which need not be a platitude. Ours is a very strenuous world; but though men by the very strenuousness of their nature may work with all their might so long as working power lasts, they nevertheless can only do the very best that is in them when full approval, by those whose approval is most authoritative, bends its cheering smile upon them. A sour want of recog nition in high quarters is fatal to high work, at any rate on a large scale. The new peerage will lighten every scientific and unscientific doctor's heart, and gladden his very often exceedingly weary mind. All of us are human enough to love approval, and to be soured by the want of it. A new impulse, a powerful impulse, will, we are certain, be given to medical study and medical work. That impulse will have the twofold effect of increasing the zeal of those who labour for the improvement of the medical art, and of hastening that difficult and laborious conquest over nature which it is the great object of science to achieve. The Marquis of Salisbury, himself distinguished in no small degree in science, has quickened the pulses of the science of the world by his recommendation of a distinguished medical man for the venerable peerage of a nation once ancient and great.

